# The Relationship between Digital Game Addiction Tendency and Depressive Symptoms in Children (36–72 Months)

**DOI:** 10.3390/children11050520

**Published:** 2024-04-26

**Authors:** Melike Yavas Celik

**Affiliations:** Department of Midwifery, Gaziantep University, Gaziantep 27310, Turkey; melikeyavascelik@gantep.edu.tr

**Keywords:** child, digital game addiction, depressive symptoms

## Abstract

**Aim:** We aimed to evaluate the relationship between digital game addiction tendency and depressive symptoms in children (36–72 months). **Method:** We conducted this research in a virtual environment with the mothers of 747 children (36–72 months). A predictive evaluation was performed using a simple regression analysis between the mean scores of the Digital Game Addiction Tendency Scale (DGATS) and the Child Depressive Symptoms Assessment Scale (CDSAS). **Results:** A total of 53.9% of children reported that they play games for 3–24 h a day. The average duration of children playing digital games was 2.86 ± 1.86 h per day. The total mean score of the CDSAS was 142.48 ± 27.36. The total mean score the children received from DGATS was 46.34 ± 17.28. In the regression analysis, it was determined that there was a strong positive correlation between the (DGATS) total score average and the (CDSAS) total score average (R = 0.52, R^2^ = 0.27, *p* < 0.05). Accordingly, digital game addiction tendency explains 27% of children’s depressive symptoms. **Conclusions:** When the standardized beta coefficient and t values are examined, it can be said that digital game addiction tendency is a significant predictor of children’s depressive symptoms.

## 1. Introduction

According to DSM-5 TR, digital game addiction can cause significant disruption or distress in a person’s life. Suggested symptoms of internet gaming disorder include preoccupation with gaming, withdrawal symptoms (sadness, anxiety, irritability, etc.) when gaming is taken away or not possible, feeling the need to spend more time playing, an inability to reduce gaming, attempting but failing to quit gaming, loss of interest in engaging in activities previously enjoyed because of gaming, continuing to play despite noticing problems, deceiving family members or others about the amount of time spent gaming, gaming to alleviate negative moods such as guilt or despair, risk, and jeopardizing or losing a job or relationship because of gaming [1].

Research on digital addiction has focused on direct effect models that investigate the relationships between psychological vulnerabilities and internet addiction. Studies have explored vulnerabilities such as depression [2], low self-esteem [3] and high adrenaline release or high thrill seeking behavior [4], loneliness and shyness [5], locus of control and online experience [6], attention-deficit/hyperactivity/impulsivity symptoms [7] and suicidal ideation [2].

With the increasing number of buildings and the decrease in playgrounds, street games played by children are gradually decreasing [8]. In addition, advances in technology and the inclusion of some technological tools in our lives have led to a mandatory digitalization process [9,10]. For this reason, street games have gradually been replaced by digital games played with digital tools such as computers, tablets, mobile phones and game consoles [11,12]. Play is the most important occupation of children. Games played in accordance with the age and development of the child have a beneficial effect on the development of the child. Ideally, a game is expected to be accessible, simple, versatile, participatory, functional and enjoyable. While playing, children acquire skills such as problem solving, exploration, thinking, reasoning, sharing, communication, strength, balance, coordination and self-organization [13,14].

It has been reported that limited access to digital games is normal, and games even have positive effects such as emotional discharge and relaxation. They have also been reported to support competencies such as following the commands given in digital games, hand–eye coordination and an improvement in motor skills. In addition, it has been stated that they support children’s problem-solving, reasoning, analytical and decision-making skills, as well as supporting their competence in strategy and prediction [15].

However, big problems can occur when digital gaming turns into addiction. Digital game addiction is defined as “the person’s excessive and compulsive use of computer or video games, even though it causes social and/or emotional problems, and the player is unable to control the excessive use” [16]. Also, studies explain the criteria for game addiction as a number of pathological behaviors such as spending excessive amounts of time in games, withdrawal, preoccupation, loss of control and interpersonal conflicts [1].

Recent studies have shown a relationship between low psychosocial well-being and an excessive or pathological use of computer and video games. Studies have determined that game addicts are less satisfied with daily life, have less self-esteem, less social competence and experience more loneliness [17]. Digital game addiction causes some behavioral changes in addicted individuals. The time these people spend in games is longer. In addition, these people cannot leave the game, are dissatisfied with life, may exhibit aggressive behavior, suffer from loneliness, have low self-esteem and have various psychological problems. Individuals who suffer from depression, attention deficits and low self-esteem are likely to become addicted to games [17].

Digital games can have more negative aspects than positive ones [18]. In the literature, especially when violent digital games are played for a long time, they have been reported to be associated with mental and psychosocial problems such as distractibility [19], loneliness [20], depression and anxiety [21], aggression [22] and violence tendency [23]. Some studies have shown that children who spend excessive time playing digital games have a decrease in academic achievement [24], inadequate and irregular sleep habits [25], insufficient physical activity [26], obesity [27] and musculoskeletal problems [28]. Physical health problems such as dryness, pain and redness in the eyes have also been reported [29].

As children get older, they become more independent, want to do things on their own and want to be more social. Children in this age group (3–6 years old) need to play with their peers in order to socialize, develop their motor skills and establish schemas. The development of motor skills and learning schemas play an important role in the growth and development process of preschool children. The addiction of this age group to digital games may cause them to be unable to learn a number of skills that affect them later in life, such as grasping and recognizing schemas, and taking steps to learn concrete operations. In this case, it may harm the academic success and social lives of these children [30]. This addiction can even lead children to depression. A study has shown that there is a low-level, positive and significant relationship between online game addictions and depression tendencies [31]. According to the results of another study, there is a significant negative relationship between online game addiction and subjective happiness [32].

Based on an accepted clinical basis that depression in children may present differently than in adults. Unlike the diagnostic criteria for adult depression, ‘masked’ symptoms may be observed in children [33]. The symptoms of masked depression seen in children were revealed for the first time by Cytryn and McKnew. These symptoms are of different types, such as school failure, aggression, somatic problems and hyperactivity [34]. The American Psychiatric Association defines childhood depression as a depressed mood or loss of interest/will, insomnia or excessive sleeping, lack of expected weight gain in children, psychomotor slowness or agitation, a child’s inability to calm themselves down, concentration problems and thoughts of worthlessness [35].

Digital game addiction has been seen as a serious problem in terms of causing behavioral problems and focus problems in children [31,32,33,34,35]. Considering the literature [18,19,20,21,22,23,24,25,26,27,28,29], it was thought that digital game addiction would be related with serious depression symptoms in young children. In addition, it was thought that by revealing the relationship between digital game addiction and depression in young children, we could attract the attention of researchers working in the field of child health and ensure further examination of this issue, which poses serious risks for children’s health. When we look at the studies conducted with older age group children (adolescents, young people and middle adolescence), it has been observed that depression and digital game addiction are related [31,36]. Therefore, it is important to evaluate how younger age groups are affected by this situation. Considering the psychological problems that children may experience, we aimed to evaluate the relationship between digital game addiction tendency and depressive symptoms in children (36–72 months).

Research Question

1. What is the relationship between digital game addiction tendency and depressive symptoms in children (36–72 Months)?

## 2. Method

The participants in this study consisted of mothers with pre-school children. Sample selection was not made in this study and all the mothers who volunteered to participate in this study and met the research criteria (familiar with the virtual environment in which the research was conducted, having a child in the preschool period (3–6 years old), having a device that could access the virtual environment where the research was conducted, loaned to the mothers in this study) were included in this study. In the end, 747 people were included in this study as the sample.

Application of research: Research is descriptive in nature. I conducted the research in a virtual environment between 23 December 2022 and 24 January 2023. The research was conducted with parents in a virtual environment using the snowball sampling method. The research materials were delivered to the participants via WhatsApp and e-mail using the Google Doc Forms program. Participants were included in the sample on a voluntary basis. The research was conducted with mothers who agreed to participate in this study. Answering the questions and scales took a maximum of 15 min. Mothers were asked to evaluate their children’s digital addiction and depression. Mothers made this evaluation based on the behaviors of their children they observed.

Data Collection Tools: Data was collected using an initial questionnaire, the Child Depressive Symptoms Assessment Scale and the Digital Game Addiction Tendency Scale.

Questionnaire: This form contained questions (questions about the child and their family) created by the researcher after the literature review [1,2,37]. The questions consisted of the mother’s age, economic status, family type, working status, number of children and their ages, and daily digital game playing time.

Digital Game Addiction Tendency Scale: This scale was developed by Budak (2020) [38]. The psychometric measurements of the scale are valid and reliable. It was determined that the item loadings of the scale were above 0.32, the correlation between the items was high and the fit values were within perfect limits. The digital game addiction tendency scale was developed in order to evaluate the digital game addiction tendencies of preschool children. It was answered by the parents. It consists of 20 items answered on a 5-point Likert scale (5: Always, 4: Often, 3: Sometimes, 2: Rarely, 1: Never). The scores obtained from the scale of 20–35 points: lowest addiction tendency; 36–51 points: low addiction tendency; 52–67 points: moderate addiction tendency; 68–83 points: high addiction tendency; 83–100 points: addiction tendency is evaluated as too much. The reliability coefficients (Cronbach’s alpha) of the sub-dimensions of the scale were as follows: Detachment from Life = 0.88, Conflict = 0.90, Continuous Play = 0.82, Projection to Life = 0.70, and the total reliability coefficient of the scale was determined as 0.93 [38]. In this study, the Cronbach alpha was found to be 0.96 for total score. Cronbach alpha coefficients for the sub-dimensions of the scale were found as follows: life = 0.87, Conflict = 0.91, Continuous Play = 0.83, and Projection to Life = 0.79.

Child Depressive Symptoms Assessment Scale: This scale was developed by Erol et al. (2020) [39]. The psychometric measurements of the scale are valid and reliable. It was determined that the item loadings of the scale were above 0.40, the correlation between the items was high and the fit values were within perfect limits. It is a five-point Likert-type scale consisting of eight sub-factors and fifty-six items developed by the researchers. It was answered by the parents. In the calculation, the ‘never’, ‘rarely’, ‘sometimes’, ‘often’ and ‘always’ options were given a value of 1, 2, 3, 4 and 5, respectively. The scale has a total of eight subscales: aggression, social adaptation, impulsivity, hyperactivity, separation anxiety, impairment in cognitive processes, somatization, incompatibility with objective reality and archaic anxiety. The scale is not applicable to children over 80 months. The Cronbach alpha coefficient of the scale was found to be 0.95 [39]. In our study, the Cronbach alpha coefficient of the scale was determined as 0.98. Cronbach alpha coefficients for the sub-dimensions of the scale were found as follows: aggression = 0.88, social adaptation = 0.87, impulsivity = 0.89, hyperactivity = 0.91, separation anxiety = 0.89, impairment in cognitive processes = 0.91, somatization = 0.88, incompatibility with objective reality = 0.88 and archaic anxiety = 0.89.

Statistical Evaluation of Data: The SPSS 23 windows package statistical analysis program was used in the analysis, creation and conclusion of the data. Number, percentage, mean and standard deviation were used as descriptive statistical methods in the evaluation of the data. In the statistical analysis, whether the numerical variables were in accordance with a normal distribution was examined with the Kolmogorov–Smirnov test and a histogram graph. It was determined that the data was normally distributed. The inter-scale correlation relationship was examined. The relationship between the scales was evaluated with Pearson correlation analysis. Regression analysis was performed due to high correlations between the scales. Also, before the regression analysis, the level of relationship was determined using correlation analysis, linearity between the variables was tested, and whether the dependent variable was at equal intervals, proportional measurement level and continuity were tested. A predictive evaluation was performed using a simple regression analysis between the mean scores of (DGATS) and (CDSAS). Scales reliability was obtained with the Cronbach Alpha test.

***Ethics of Research:*** Ethics committee approval was obtained from the Ethics Committee of a University (Reference no: 2022/429/7 December 2022). Written and verbal consent was obtained from the mothers. The research was conducted in accordance with the principles of the Declaration of Helsinki.

## 3. Results

In this study, of the mothers, 52.2% were 26–35 years old, 89% lived in a nuclear family, 55.2% had completed undergraduate education or had received postgraduate education, 52.2% had an income proportional to their expenses, 45.5% had two or three children. Of children, 61.3% were between 49 and 72 months. A total of 53.9% of the children reported that they play games for 3–24 h a day. The average duration of children playing digital games was 2.86 ± 1.86 h per day (Table 1).

The total mean score the children received from DGATS was 46.34 ± 17.28. The average scores the children received from the subscales of DGATS were as follows: Detachment from Life 14.31 ± 6.08, Conflict 10.11 ± 4.11, Constantly Playing 14.46 ± 5.61 and Projection to Life 7.47 ± 3.01 (Table 2).

The total mean score of CDSAS was 142.48 ± 27.36. The average scores the children received from subscales of CDSAS were as follows: aggressions 14.09 ± 5.56, social adaptation 33.47 ± 6.21, impulsivity–hyperactivity 17.66 ± 4.32, separation anxiety 13.64 ± 3.58, impairment in cognitive processes 25.56 ± 7.76, somatization 11.33 ± 3.16, incompatibility with objective reality 18.23 ± 6.05 and archaic anxiety 142.48 ± 27.36 (Table 3).

It was determined that there was a positive and strong correlation between the total score averages of DGATS and CDSAS (R = 0.52, R^2^ = 0.27, *p* < 0.05). Accordingly, digital game addiction tendency explains 27% of children’s depressive symptoms. In addition, a strong correlation was found between the scores of the DGATS sub-dimensions and the total scoring of CDSAS (Detached from Life: R = 0.50, R^2^ = 0.25, *p* < 0.05; Conflict: R = 0.47, R^2^ = 0.22, *p* < 0.05; Continuous Playing: R = 0.45, R^2^ = 0.21, *p* < 0.05; and Projection to Life: R = 0.47, R^2^ = 0.22, *p* < 0.05) (Table 4). Accordingly, when the standardized beta coefficient and t values are examined, it can be said that digital game addiction tendencies are a significant predictor of children’s depressive symptoms.

## 4. Discussions

Online games, which can be played online individually or by multiple users, have become playable at any time and place on various devices, such as mobile devices other than computers and game consoles, due to the development of technology. The development of online games has made it possible to interact with others by eliminating the necessity of being physically together with another person. The emergence of massive multiplayer online games has enabled players to explore new worlds and brought individuals together for digital companionship. It has been claimed that this social aspect provided by online games increases many people’s addiction to online games [40]. In this study, in which we aimed to evaluate the effect of digital game addiction tendencies of preschool children (36–72 months) on children’s depressive symptoms, we determined that children’s addiction rates are quite high. In this study, it was determined that the majority of children played games for 3 h or more, and that the daily playing time of children was long enough to harm their health. It was also observed that the average scores of children on the Digital Game Addiction Tendency scale were also high [38]. It has been determined that children in early childhood spend an average of 2 h a day playing these games, where their digital game preferences increase [10,37,41]. Playing digital games for a long time worries experts and parents. Unconsciously, and without being bound by certain rules, playing digital games increases the tendency to these games and causes negative effects on children due to excessive exposure [42]. It has been determined that playing digital games excessively can cause addiction in children [43].

Studies have shown that digital game addiction may be associated with problematic behaviors and aggression in children [44,45,46,47,48]. In addition, in a study investigating the negative effects of digital games on children’s mental and physical health, it was stated that the average age of starting to play digital games is 1–6 years old, and it has been reported that the negative effects of digital games include digital game addiction, the effect violent games have on children’s moods, depression, lack of communication within the family, posture disorder and sleep quality disorders [29]. In another study examining the aggression, depression and loneliness levels of primary school students who play and do not play computer games, it was concluded that as the duration of playing computer games increased, anti-social aggression increased, and violent computer games affected the emergence of anti-social behaviors [45]. Unlike the diagnostic criteria for adult depression, ‘masked’ symptoms can be observed in children [33]. The concept of masked depression, first described by Kielholz, was adapted for children by Cytryn and McKnew. They added different symptoms such as school failure, aggression, somatic problems and hyperactivity to the depression clinic [34]. In addition to introverted symptoms such as anxiety, fear, attention problems, reluctance, lack of pleasure, lethargy and inhibition, the relationship between extroverted symptoms, such as aggression, hyperactivity, impulsivity, agitation, or behavioral symptoms such as sleep, overeating and social adaptation problems, with depression have been mentioned [33,49,50]. These symptoms negatively affect the growth and development of children and prevent them from socializing. It is therefore a useful initiative to identify conditions that may affect the symptoms of depression in children. Digital game addiction, which is becoming increasingly common nowadays, has also been shown in this study to be a strong predictor of depression symptoms in children. In addition, the average score of these children with moderate digital game addiction on CDSAS, which evaluates their depression levels, was determined to be 142.48 ± 27.36. It has been determined that playing digital games causes psychological problems such as anxiety and aggressive attitudes (74.8%), and depression and asocialization (69.7%) in children [29]. In a study conducted with high school students, a low-level positive relationship was determined between game addiction and depression, and between game addiction and loneliness [31]. In the study conducted by Brunborg et al. (2018) with 1928 high school students, a significant relationship was found between the students’ digital game addiction levels and the students’ perceived depression [51]. According to another study conducted by Mehroof and Griiths (2010), participants stated that their anxiety levels increased as the time they spent with digital games increased [52]. Sancaktar (2020) stated that there was a significant relationship between the time adolescents play digital games and the level of stress they experience and perceived social support [53].

As can be seen, there is a strong relationship between depression and digital game addiction. It is extremely important to undertake initiatives to solve this problem, in order to ensure that children receive the necessary psychosocial support and to cooperate with the family. For this reason, individuals and parents of young children who are addicted must be informed urgently and these children must be saved from digital game addiction. But unfortunately, research shows that parents have an average knowledge of digital game outcomes [54,55]. In this process, parents did not know exactly what to do, were worried and tended to wrong practices such as punishment [56]. Determining the guidance strategy that parents use when their children play digital games can make this process more accurate [57]. Parents play a critical role in the lives of preschool children [58]. If the parent thinks that the digital game harms their child, it is necessary to learn the level of addiction tendency of the child with excessive and inappropriate use, to take measures accordingly, and to establish the child’s digital life balance [59]. It is important to determine the level of children’s use of digital games in order to intervene in children with a high tendency to addiction. The content of the behavior of parents, which is considered among the most important factors of the digital game process, during the digital interaction of their children is also important. In this process, the parent should observe their child, control the content of the game and be in contact with them [60,61].

Also, depressive symptoms in this children should not be ignored and psychological support should be sought if necessary. Aggression is seen at a high rate in children addicted to digital games. Such children are not accepted by their peers, and negative attitudes such as being more introverted and showing more behavioral problems are more common in these children. Unfortunately, the development of these children who do not play with their peers and are left alone is negatively affected. Due to their inability to socialize and their increasing introversion, these children unfortunately experience problems such as having fun with digital games, confusing their dream world with the real world and experiencing everything as quickly as in the digital world. Unfortunately, it is not possible for this age group to cope with all these problems alone. When it comes to young children, there are also undesirable coping mechanisms [2,3,4,5,18,25]. For example, coping mechanisms such as regression, fantasy and repression may occur. Even negative situations such as depression and suicidal thoughts may occur [30]. These children have been living in the digital world for so long that, unfortunately, they may not be able to keep up with life in the real world [58,60]. For this reason, these children should not be ignored and they should receive help overcoming this addiction through support from experts.

## 5. Conclusions

In conclusion, it was determined in our study that preschool children’s digital game addiction is approximately moderate and digital game addiction tendency is associated with children’s depression symptoms. It was also found that children’s digital game addiction was moderate and their depression levels were high.

Therefore, it can be said that digital game addiction tendency is a strong predictor of children’s depression symptoms, and a tendency for digital games may increase their depression symptoms. Considering the increasing digital addiction in children today, it can be predicted how great the danger awaiting children and their parents can be. It can be said that parents have an important place in preventing this danger for pre-school children and parents should be supported to develop a guidance strategy that their children use while playing digital games. Parents should be aware of the types of play their children prefer and replace the feeling of pleasure of playing digital games, or even playing at a risky level, with the right alternative activities. It would be useless to impose restrictions and remove computers and digital devices to keep the child away from digital play. For this reason, determining the types of games that children like and directing them to active sports can prevent this addiction.

In future studies, the relationship between children’s cyberbullying or victimization and their game addictions should be examined. It is especially important for parents and nurses to be conscious and aware of the digital world in guiding students.

This study, which we think will provide a basis for future studies, will encourage researchers to examine the problems underlying this relationship between depression and digital game addiction. It can be a resource for providing new practices, training and initiatives prepared by healthcare professionals to protect child mental health.

### Limitation of Research

The fact that this research was conducted on a sample covering a certain age group and was conducted via social media constitutes a limitation. Another limitation of this study is that no attempt was made to explain the reason behind the relationship between digital game addiction and depression.

## Figures and Tables

**Table 1:** Demographic data of children and their mothers.

Data		n = 747	%
Age of mother	18–25	218	29.2
	26–35	390	52.2
	36–55	139	18.6
Type of Family	Nuclear	665	89.0
	Extended	82	11.0
Education status	Literate/illiterate or primary school graduate	118	15.8
	Middle school or high school	217	29.0
	Undergraduate or higher education	412	55.2
Mother’s working status	Yes	289	38.7
	No	458	61.3
Income status	Income less than expenses	229	30.7
	Income balanced with expenses	390	52.2
	Income more than expenses	128	17.1
Number of children	1 child	307	41.1
	2–3 children	340	45.5
	4–10 children	100	13.4
Child’s age	36–48 months	289	38.7
	49–72 months	458	61.3
The duration of children playing digital games per day	1–2 h	344	46.1
	3–24 h	403	53.9
The average duration of children playing digital games per day	2.86 ± 1.86		

**Table 2:** Average scores of children from total DGATS and subscales.

DGATS and Subscales	Mean ± SD	Min–Max
Detachment from Life	14.31 ± 6.08	13.87–14.74
Conflict	10.11 ± 4.11	9.81–10.41
Continuous Playing	14.46 ± 5.61	14.05–14.86
Projection to life	7.47 ± 3.01	7.25–7.65
Total DGATS	46.34 ± 17.28	45.10–47.59

**Table 3:** Average scores of children from total CDSAS and subscales.

CDSAS and Subscales	Mean ± SD	Min–Max
Aggression	14.09 ± 5.56	13.69–14.49
Social adaptation	33.47 ± 6.21	33.02–33.92
Impulsivity–hyperactivity	17.66 ± 4.32	17.35–17.97
Separation anxiety	13.64 ± 3.58	13.38–13.91
Impairment in cognitive processes	25.56 ± 7.76	25.01–26.12
Somatization	11.33 ± 3.16	11.10–11.55
Incompatibility with objective reality	18.23 ± 6.05	17.81–18.67
Archaic anxiety	8.46 ± 2.63	8.27–8.65
Total CDSAS	142.48 ± 27.36	140.51–144.45

**Table 4:** The effect of preschool children’s digital game addiction tendencies on children’s depressive symptoms.

Independent Variables (DGATS)	Dependent Variables = Child Depressive Symptoms Assessment Scale (CDSAS)								
	Mean ± S.D	F **/*p*	R	R^2^ *	(r)/*p*	t/*p*	Durbin–Watson	B	Beta
Detachment from Life	14.31 ± 6.08	243.32/0.01	0.50	0.25	0.50/0.01	48.40/0.01	1.85	2.23	0.50
Conflict	10.11 ± 4.10	207.31/0.01	0.47	0.22	0.47/0.01	45.83/0.01	1.89	3.11	0.47
Continuous Playing	14.46 ± 5.60	190.58/0.01	0.45	0.21	0.45/0.01	43.36/0.01	1.83	2.21	0.45
Projection to Life	07.47 ± 3.01	211.91/0.01	0.47	0.22	0.47/0.01	45.52/0.01	1.85	4.28	0.47
DGATS total	46.35 ± 17.28	264.50/0.01	0.52	0.27	0.52/0.01	41.20/0.01	1.85	0.81	0.52

* Simple regression analysis, ** ANOVA.

## Data Availability

The original contributions presented in the study are included in the article, further inquiries can be directed to the corresponding author.
